# Evaluation of narrative nursing combined with motivational care in improving emotional status and reducing anxiety among chronic renal failure patients receiving hemodialysis

**DOI:** 10.1097/MD.0000000000047779

**Published:** 2026-07-10

**Authors:** Ke Mei, Jun Gao, Ziqun Peng, Ziang Zhang, Guohui Liu

**Affiliations:** a The Banan Affiliated Hospital of Chongqing Medical University, Chongqing City, China; b The Banan District Jielong Central Hospital of Chongqing, Chongqing City, China; c Chongqing Medical University, Chongqing City, China.

**Keywords:** anxiety, chronic renal failure, hemodialysis, motivational, narrative nursing, psychological well-being

## Abstract

This study aimed to explore the effects of integrating narrative nursing with motivational interviewing on treatment adherence and anxiety among patients with chronic renal failure (CRF) undergoing hemodialysis. From April 2023 to April 2024, according to different treatment methods, a total of 76 patients receiving maintenance hemodialysis for CRF were divided into either a control group, which received routine nursing care, or an observation group that received a combination of narrative and motivational nursing in addition to standard care. The psychological and behavioral outcomes were evaluated using the Hamilton Anxiety Scale (HAMA), Hamilton Depression Scale (HAMD), Medical Coping Modes Questionnaire, and General Quality of Life Inventory-74 (GQOLI-74). At baseline, no significant differences were observed in HAMA and HAMD scores between the 3 groups. Following the nursing intervention, both groups exhibited significant declines in anxiety and depression scores, with the observation group showing more pronounced improvements. Furthermore, patients in the observation group demonstrated more positive coping patterns, higher quality of life scores, and greater satisfaction with nursing care. Pearson correlation and receiver operating characteristic analyses confirmed that the combined intervention effectively improved emotional regulation and compliance, showing high sensitivity and specificity. The integration of narrative and motivational nursing markedly mitigates negative emotional states, enhances self-management and engagement, and improves overall nursing satisfaction among CRF patients on hemodialysis. These findings highlight its clinical value and support wider application in comprehensive renal care.

## 1. Introduction

Chronic renal failure (CRF) represents the progressive outcome of various chronic kidney diseases, clinically manifested by renal insufficiency, retention of metabolic waste products, an imbalanced internal environment, and dysfunction across multiple systems.^[1]^ Clinical statistics reveal an annual incidence rate of approximately 0.3‰ for CRF, displaying an upward trend over the years. In China, the prevalence of chronic kidney diseases among adults is estimated to be around 10.8%.^[2]^ The clinical symptoms of patients with chronic kidney failure are exceedingly complex, categorizable into 2 major classes: metabolic abnormalities and systemic multisystemic symptoms. The majority of patients in stages I–III are either asymptomatic or exhibit mild symptoms such as fatigue, anorexia, and mild anemia.^[3]^ As the condition progresses, patients may experience complications including congestive heart failure, hyperkalemia, gastrointestinal symptoms, and subsequent occurrences of bleeding and neurological disorders, posing significant threats to their health.^[4]^ Hemodialysis stands as a renal replacement therapy specifically designed for CRF patients, effectively eliminating metabolic and toxic substances from the body, maintaining electrolyte and acid-base balance, and removing excess body fluids, thus significantly ameliorating clinical symptoms and prognosis.^[5]^ According to pertinent research findings, hemodialysis alleviates symptoms and enhances quality of life within a 6-week treatment period. However, this condition is prone to recurrence, and long-term hemodialysis may lead to physical discomfort.^[6]^ Patients often experience emotions such as anxiety and depression, severely compromising the efficacy of hemodialysis treatment. Therapeutic strategies are centered around correcting risk factors and preventing complications.

With the rapid advancement of surgical techniques, interdisciplinary collaboration has become imperative within healthcare settings. Consequently, there has been a gradual escalation in the requirements for nurses and their nursing capabilities.^[7]^ Therefore, nurses of the future are expected to possess exemplary critical thinking skills and clinical proficiency.^[8]^ Narrative nursing stands as a nursing approach whose effectiveness and feasibility have been validated in clinical practice. Particularly within the treatment milieu of hemodialysis, this modality emphasizes the differentiation between the patient’s current ailments and issues from their identity.^[9]^ Through narrative nursing, patients are encouraged to unearth daily pleasures and personal strengths within the narratives of their life stories, utilizing these positive and healthy elements to construct subplots, thereby aiding in the redirection of attention away from the main narrative focus on illness and treatment. Employing a narrative approach not only prompts patients to share their personal experiences more actively but also aids in the alleviation of their inner turmoil.^[10]^ Throughout this process, nursing staff can promptly detect changes in the patient’s condition and psychological state, effectively engaging in emotional guidance and problem-solving. This method, through reshaping the framework of the patient’s story, enhances patient engagement and the overall effectiveness of treatment.^[11]^ Motivational nursing encompasses meticulous patient assessment, preoperative preparation, and postoperative monitoring. Nurses play a pivotal role in ensuring procedural safety and minimizing risks through vigilant observation and adherence to evidence-based guidelines. Furthermore, motivational nursing underscores interdisciplinary collaboration, particularly in complex nursing requiring coordinated efforts among healthcare providers.^[12]^ This collaborative approach enhances procedural efficiency and patient safety, underscoring the indispensable role of nursing within the broader healthcare team.

Effective nursing has been shown to reduce healthcare costs and increase patient satisfaction. Additionally, research indicates that competent nursing care during hemodialysis contributes to disease recovery and improves patient prognosis. Traditional health education formats are often limited in scope and lack comprehensive content, resulting in suboptimal overall efficacy and inadequacy in meeting clinical needs.^[13]^ Motivational nursing represents a widely utilized high-quality nursing service ensuring comprehensive, dynamic, and continuous specialized care, which has been applied across various diseases such as stroke and acute coronary syndrome. When combined with narrative nursing, this approach facilitates the regulation of negative emotions in patients while enhancing their confidence in diagnosis and treatment, thus serving as a crucial avenue for unlocking patients’ intrinsic potential. Therefore, CRF patients undergoing hemodialysis at our institution were selected to analyze the effects of narrative nursing combined with motivational nursing on compliance and anxiety during hemodialysis. This integrated nursing approach aims to optimize patient care outcomes and improve the overall quality of care provided to CRF patients undergoing hemodialysis.

## 2. Materials and methods

### 2.1. Study design and population

This study was approved by the Ethics Committee of The Banan Affiliated Hospital of Chongqing Medical University. The study involved a retrospective analysis of CRF patients undergoing hemodialysis from April 2023 to April 2024 in our hospital. Prior to enrollment, all patients provided informed consent and signed consent forms. All procedures adhered to the ethical principles outlined in the Helsinki Declaration for clinical research, and ethical approval of the study protocol was obtained from the ethics committee of our hospital. Inclusion criteria: patients meeting the diagnostic criteria for end-stage renal disease outlined in the “Guidelines for Diagnosis and Treatment of Chronic Kidney Failure,” with a glomerular filtration rate [(140 − age) × weight (kg)/serum creatinine (μmol/L), multiplied by a factor of 1.23 for males and 1.04 for females] declining to ≤15 mL/(min·1.73m^2^). Patients with a 24-hour urine output of <400 mL, oliguria, or anuria, with fluid intake < total daily urine output + 500 mL. Patients undergoing regular dialysis for ≥1.5 months. Patients classified as stage IV and V chronic kidney disease. Exclusion criteria: Patients receiving treatment that may affect the results of this study within 0.5 months prior to admission. Patients with factors contributing to worsening renal function within the past 2 months, such as congestive heart failure. Patients with severe heart or liver diseases, such as cirrhosis or coronary heart disease.

### 2.2. Treatment methods

All patients underwent hemodialysis therapy. Hemodialysis was performed using polysulfone high-flux dialyzers from B.Braun Avitum AG under systemic heparinization, with a dialysate flow rate of 500 mL/min and blood flow rates of 200 to 300 mL/min, for 4 hours per session, 3 times per week.

In the conventional nursing group (control group), nursing care primarily encompassed providing a conducive therapeutic environment, medication guidance, and monitoring of the treatment process. The specific implementation was as follows: First, ensuring the creation of a relatively comfortable treatment environment for the patients, meeting their reasonable needs as much as possible, and fully respecting their privacy during the nursing process. Second, providing medication guidance in accordance with the physician’s instructions while reminding family members to enhance their companionship with the patient, thereby offering emotional support to alleviate the patient’s psychological burden. In terms of treatment monitoring, the nursing team continuously monitored the patients’ physiological parameters. Any abnormalities detected must be promptly reported to the attending physician for timely adjustment of the treatment strategy to ensure patient safety and treatment efficacy. This comprehensive nursing strategy aims to optimize the patient’s treatment experience and improve treatment outcomes.

In the Observation Group, we have implemented motivational psychological nursing. Preexamination involved, first, clarification of the Treatment Plan: During the initial hospitalization phase, medical personnel collected and organized general patient information to create personal files (including etiology, disease duration, treatment plans, etc). Through communication with the patients, their treatment needs and personal cognitive abilities were clarified. Analysis was conducted based on the principles of protection motivation theory (susceptibility, severity, external and internal factors) and coping factors (self-efficacy, response efficacy, and response cost) to devise targeted plans. The importance of fostering a good doctor-patient relationship was emphasized to the patients, and nursing measures were promptly adjusted based on changes in their condition and personal preferences. Second, information delivery (weeks 1–2): Objective of protection motivation: during this period, efforts were made to enhance MHD patients’ understanding of disease susceptibility and severity. Protection motivation measures: within 5 days prior to admission, medical personnel intensified communication with patients and their families to extensively disseminate relevant health knowledge regarding uremia, including etiology, symptoms, diagnostic methods, treatment options, and prognosis. Patients were educated about the dangers of noncompliance with medical advice and the impact of unhealthy lifestyle habits on treatment. In the second week, medical personnel utilized books and other materials to enhance patients’ awareness of the hazards of the disease. Short videos showcasing successful treatment cases were also played to increase patients’ susceptibility. The frequency was 60 minutes per session, once a week. Last, conducting health lectures (weeks 3–7) – Objective of protection motivation: this period aimed to gradually improve external and internal factors affecting patients’ treatment outcomes. Protection motivation measures – During Examination: Due to unfamiliarity with the environment or lack of understanding of the diagnostic procedures, patients commonly experience a sense of unfamiliarity upon entering the examination room. Therefore, nursing staff provided verbal or nonverbal encouragement to help patients build confidence. After Examination: nursing staff patiently listened to patients’ nursing needs and positive experiences during the examination, actively addressing any deficiencies in the examination process for improvement.

Additionally, we supplement our care with narrative nursing. In the observation group, narrative nursing was implemented in addition to routine nursing care, while the control group received standard nursing care. Narrative nursing comprised 6 stages: preparation, plan formulation, externalizing problems, deconstructing problems, rewriting problems, and storytelling witness. During the preparation stage, nursing personnel conducted comprehensive and systematic patient assessments to gain insight into the patient’s condition and establish a rapport to earn the patient’s trust. Nursing staff focused on the patient’s psychological state and emotional fluctuations, patiently listened to and observed the patient’s words and actions, recorded key information, encouraged the patient to express freely, ensured timely response to their needs, and demonstrated support and affirmation. For hemodialysis patients, considering their typically thrice-weekly dialysis sessions, narrative nursing sessions were scheduled on dialysis days during the patient’s hospitalization. Narrative nursing sessions were conducted 3 times a week, lasting 10 to 20 minutes each time. Moreover, with unbiased support, patients’ narratives were respected, affirming each patient as the independent “expert” of their condition. Nursing staff positively acknowledged and respected patients’ narratives when sharing their condition and dialysis treatment experiences, encouraging further expression to help them construct psychological images. By integrating the patient’s previous narrative content, patients were guided to recall how they coped with challenges in the past, assisting them in recognizing their problem-solving process to enhance their sense of problem-solving experience. Narrative records were analyzed, and based on the specific circumstances, negative content within the stories was adjusted, and blueprints for actions and meanings were designed to assist patients in reshaping themselves. Support was provided for their psychological preparation during the treatment process, alongside providing specific self-management tools. Building upon the original narrative content, narratives were reconstructed to enhance the patient’s confidence and assist them in viewing themselves from a new perspective, while also organizing personal experiences. With patient consent, family members were invited to witness the patient’s efforts during treatment and recovery, providing affirmation. This not only boosted the patient’s treatment confidence but also facilitated more effective support from family members. These narrative nursing sessions were conducted during dialysis sessions, lasting for 4 weeks, with each session lasting 20 to 30 minutes.

### 2.3. Observation indicators

Psychological resilience assessment was conducted using the Connor-Davidson Resilience Scale (CD-RISC). The scale is divided into 3 dimensions: resilience, optimism, and self-improvement, with scores ranging from 0 to 100. Higher scores indicate better psychological resilience. Hope level: hope level assessment was performed using the Herth Hope Index (HHI). The scale comprises 3 dimensions: positive attitude, positive behavior, and maintaining close relationships with others, with scores ranging from 12 to 48. Higher scores indicate higher hope levels.

Before and after nursing, the Medical Coping Modes Questionnaire was utilized to assess disease coping methods. The scale’s Cronbach’s α is 0.793, comprising yielding (20 points), avoidance (28 points), and confronting (32 points). Higher scores indicate a tendency towards the respective coping method.

The Short Form Health Survey (SF-36) was used to evaluate quality of life, covering physical, emotional, role, social, and cognitive functions, with each dimension scored out of 100. Higher scores indicate better quality of life.

The Hamilton Depression Scale (HAMD) and Anxiety Scale (HAMA) were used to assess patients’ adverse emotional states. Scores below 17 are considered normal, and lower scores correlate negatively with patients’ adverse emotions, indicating more significant improvements in emotional state.

Nursing effects were evaluated from 4 aspects: compliance behavior, emotional stability, knowledge, and self-care.

### 2.4. Statistical methods

SPSS 23.0 software (SPSS Inc., Chicago) was used for analysis. Descriptive statistics were presented as mean ± standard deviation. Between-group and within-group comparisons were conducted using *t*-tests. Pearson correlation analysis and receiver operating characteristic (ROC) curve analysis were employed to assess the relationship between psychological status indicators (HAMD and HAMA scores). *P* < .05 was considered statistically significant.

## 3. Results

### 3.1. Demographic characteristics

From April 2023 to April 2024, a total of 76 patients diagnosed with CRF were divided into 2 groups for comparative analysis. There were no significant differences in baseline characteristics between the 2 groups (Table 1).

**Table 1:** Comparison of general information.

Characteristics	Observation group (n = 41)	Control group (n = 35)	*t*	*P*
Age	55.6 ± 6.78	56 ± 5.90	−0.315	.754
Course of disease (mo)	18.44 ± 3.84	18.6 ± 3.97	−0.205	.838
Income	3056.5 ± 225.65	3068.84 ± 218.70	−0.278	.782

### 3.2. Effectiveness analysis

Before nursing, there was no statistically significant difference in CD-RISC and HHI scores between the 2 groups (*P* > .05). After 8 weeks of nursing, the CD-RISC and HHI scores in the study group were higher than those in the control group, with statistically significant differences (*P* < .05). The quality of life indicators, including physical function, role functions, social function, and cognitive function, in the Observation group were all better after nursing (*P* < .05). Moreover, the HAMA and HAMD scores in the Observation group were significantly lower than those in the Control group, with statistically significant differences (both *P* < .05; Table 2).

**Table 2:** Comparison of relevant indicators before and after nursing intervention.

Parameter	Observation group (n = 41)	Control group (n = 35)	*t*	*P*
CD-RISC				
Before treatment	55.48 ± 4.82	55.6 ± 4.55	−0.128	.898
After treatment	74.04 ± 5.20*†	63.48 ± 6.67	8.83	.000
HHI				
Before treatment	25.08 ± 3.29	25.2 ± 3.18	-0.185	.853
After treatment	38.44 ± 4.01*†	32.52 ± 3.50	7.869	.000
Physical function				
Before treatment	45.34 ± 4.77	46.24 ± 5.42	−0.881	.38
After treatment	70.4 ± 6.27*†	56.2 ± 6.42	11.189	.000
Role functions				
Before treatment	48.46 ± 7.72	47.64 ± 4.23	0.659	.511
After treatment	68.64 ± 7.20*†	54.32 ± 7.21	9.937	.000
Function of society				
Before treatment	51.26 ± 4.77	50.76 ± 4.49	0.539	.591
After treatment	65.46 ± 6.25*†	58.46 ± 5.26	6.06	.000
Cognitive function				
Before treatment	55.86 ± 6.40	54.64 ± 5.26	1.042	.3
After treatment	75.56 ± 7.25*†	59.46 ± 4.22	13.574	.000
HAMA Scores				
Before treatment	23.26 ± 2.52	23.48 ± 2.49	−0.439	.661
After treatment	10.26 ± 1.32*†	15.48 ± 1.79	−16.606	.000
HAMD Scores				
Before treatment	25.58 ± 2.30	25.48 ± 2.45	0.21	.834
After treatment	11.44 ± 1.39*†	17.48 ± 1.93	−17.97	.000

CD-RISC = Connor-Davidson Resilience Scale, HAMA = Hamilton Anxiety Scale, HAMD = Hamilton Depression Scale, HHI = Herth Hope Index.

* indicates comparison with the same group before treatment, *P* < .05.

† indicates comparison with the Control group after treatment, *P* < .05.

### 3.3. Complications

Both the Medical Coping Modes Questionnaire scores and GQOLI-74 scores before and after nursing were better in the Observation group, as shown in Table 3.

**Table 3:** Comparison of MCMQ Scores and GQOLI-74 Scores before and after nursing intervention in both groups.

Parameter	Observation group (n = 41)	Control group (n = 35)	*t*	*P*
Give in to				
Before treatment	13.08 ± 2.16	13.04 ± 2.12	0.094	.926
After treatment	7.68 ± 0.96*†	11.86 ± 1.13	−20.011	.000
Avoidance				
Before treatment	18.24 ± 2.67	18.22 ± 2.64	0.038	.97
After treatment	10.28 ± 1.11*†	15.00 ± 1.37	−18.945	.000
Face to face				
Before treatment	17.38 ± 3.04	17.42 ± 3.02	−0.066	.947
After treatment	23.34 ± 2.18*†	19.30 ± 2.04	9.558	.000
GQOLI-74				
Before treatment	158.20 ± 8.70	158.28 ± 8.73	−0.046	.963
After treatment	304.54 ± 12.67*†	263.88 ± 10.58	17.413	.000

GQOLI-74 = General Quality of Life Inventory-74, MCMQ = Medical Coping Modes Questionnaire.

* indicates comparison with the same group before treatment, *P* < .05.

† indicates comparison with the Control group after treatment, *P* < .05.

### 3.4. ROC evaluation of renal function indicators

**Figure 1.**
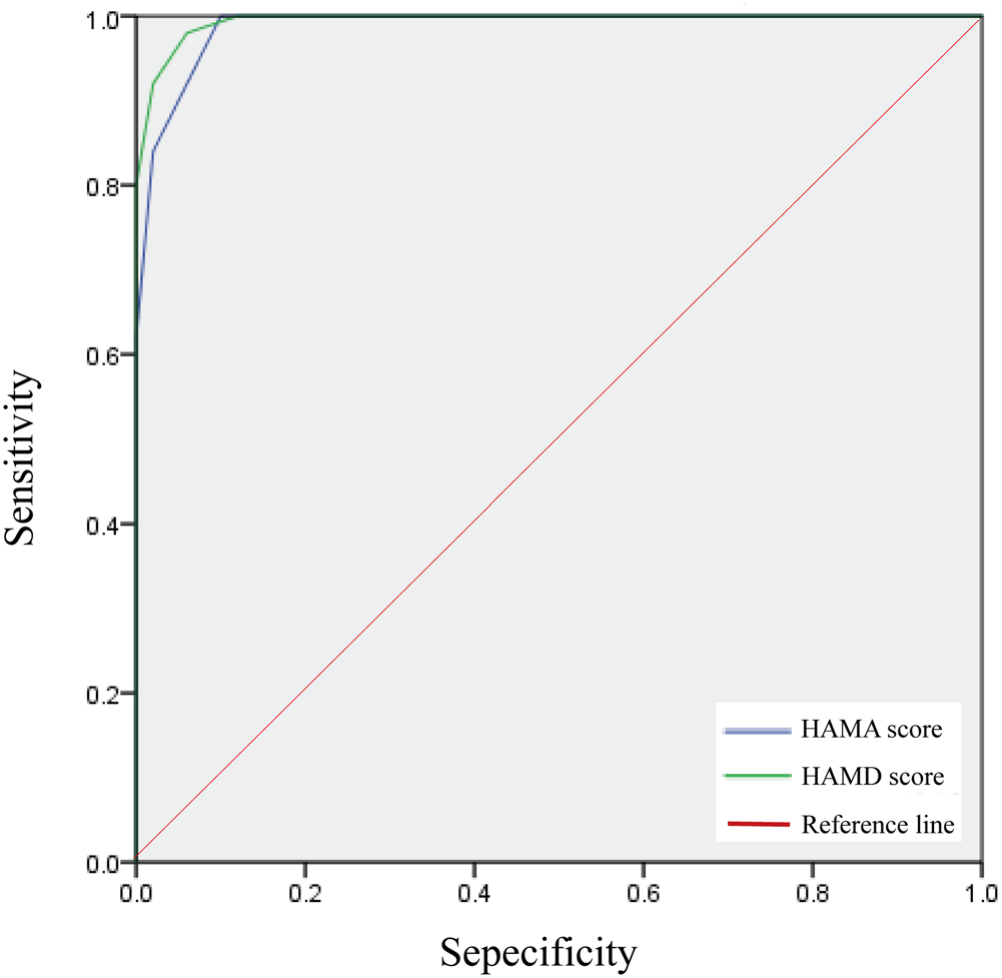
ROC curve analysis of psychological status indicators predicting the prognosis of CRF patients, including the Hamilton Anxiety Scale (HAMA) and Hamilton Depression Scale (HAMD) scores. HAMA = Hamilton Anxiety Scale, HAMD = Hamilton Depression Scale.

In this study, ROC curve analysis was used to assess the value of psychological status indicators in predicting the prognosis of CRF patients under structured nursing. The results showed that the cutoff value of the HAMA score was 13.5 points, with a sensitivity and specificity of 100% and 90%, respectively. The cutoff value of the HAMD score was 14.5 points, with a sensitivity and specificity of 98% and 94%, respectively. The AUC for both was 0.988 and 0.995, respectively, as detailed in Figure 1.

## 4. Discussion

CRF manifests as a syndrome characterized by metabolic disturbances and multifaceted symptoms due to advanced kidney disease. The chronicity and severity of CRF necessitate long-term hemodialysis, imposing substantial physical and psychological burdens on patients.^[14]^ Hemodialysis is a critical nursing intervention for prolonging survival in end-stage renal disease, establishing extracorporeal circulation via hemodialysis equipment. Blood and dialysate flow into the dialyzer, where the semipermeable membrane facilitates the diffusion of harmful substances and metabolic waste from the blood, correcting electrolyte imbalances, fluid overload, and acid-base disturbances.^[15]^ However, the extended duration of hemodialysis, coupled with dialysis-related complications and economic pressures, often exacerbates patients’ psychological distress, leading to reduced treatment confidences.^[16]^ Persistent psychological issues not only diminish the quality of life for hemodialysis patients but also disrupt adherence to medical recommendations, thereby compromising treatment efficacy. Conventional nursing models typically involve unilateral health education, focusing solely on the transmission of health knowledge through formalized nursing.^[17]^ As a result, patients may not actively engage in learning health-related information, and their compliance with medical advice may be overlooked, potentially leading to psychological resistance and resentment.^[18]^ These challenges significantly hinder the effectiveness of health education. Conversely, group health education involves assembling patients with similar conditions to implement collective health management strategies.^[19]^ This approach can enhance motivation for learning, improve the effectiveness of health education, and foster mutual support and encouragement among patients.^[20]^

The results of this study demonstrate that after nursing, the observation group exhibited significantly lower HAMA and HAMD scores compared to the control group (*P* < .01), indicating that the integration of motivational psychological care and narrative therapy effectively alleviates anxiety and depressive symptoms in hemodialysis patients. Additionally, quality of life indicators (physical, emotional, role, social, and cognitive functions) were generally superior in the observation group. The efficacy of these outcomes can be attributed to the critical role of psychological care in clinical nursing. Motivational psychological care, grounded in psychological theories, addresses various aspects of patient psychology, activities, and characteristics, guiding patient care through specific plans and steps.^[21]^ During this care, patients’ emotional states are assessed by listening to their concerns regarding the catheterization procedure and their condition.^[22]^ Discussing the catheterization process helps patients understand its necessity and purpose. Encouragement and positive persuasion alleviate negative emotions, stimulate patients’ potential and positive emotions, and enhance their subjective initiative and self-control, enabling them to face catheterization with a positive mindset. Narrative therapy, rooted in narrative medicine, represents a novel nursing model. By employing storytelling, internal problem externalization, problem deconstruction, and rewriting, it provides psychological support.^[23]^ This approach helps patients shift their thinking patterns, ignite self-identification, reconstruct values and life’s meaning, correct negative emotions, and strengthen their survival beliefs.^[24]^ Narrative therapy not only offers an emotional release channel but also aids nurses in understanding patients’ moods and psychological support needs. Positive guidance from nurses stimulates patients’ potential, helping them overcome psychological challenges and fostering recovery confidence.^[25]^ Psychological resilience, a flexible dynamic form, reflects patients’ behavioral and psychological responses to external changes. This indicates that motivational psychological care can alter hemodialysis patients’ coping strategies, enabling them to face their illness with a positive attitude.

Combining motivational psychological nursing with narrative nursing represents a departure from traditional, non-standardized practices, allowing nursing staff to move beyond subjective habits and experiences. This integration enhances the fluidity of the nursing process, crucially improving the quality of care and contributing to examination efficiency and safety.^[26]^ Motivational psychological nursing, when combined with procedural nursing, provides significant psychological support, stimulating motivation, setting goals, increasing patient engagement, and mobilizing their inner potential. This ensures comprehensive nursing guidance. Narrative nursing centers the patient in service delivery, using storytelling to help patients articulate their negative emotions and alleviate self-perceived burdens.^[27]^ Nursing staff must respect each patient’s story, listening attentively with equality and respect, and encouraging patients to share their dialysis experiences.^[28]^ This approach makes patients feel valued and cared for, allowing them to express negative emotions freely, thereby strengthening the nurse-patient relationship and dispelling psychological concerns.^[29]^ The advantages of combining motivational psychological nursing with narrative nursing are more pronounced than using single nursing methods. Our analysis of patients’ negative emotions, mental health, and quality of life confirmed that combined nursing better improves patients’ self-care abilities. Motivational psychological nursing boosts patients’ confidence through encouraging measures and targeted addressing influencing factors.^[30]^ Emotions significantly impact disease examination and treatment, potentially interfering with outcomes. Thus, assessing the psychological status of hospitalized patients and providing targeted psychological support and health education based on specific conditions is essential.^[31]^ This study has several limitations. First, it was conducted at a single center, which may limit the generalizability of the findings. Second, the sample size was relatively small, potentially reducing statistical power. Third, due to the retrospective design and relatively short observation period, long-term outcomes could not be evaluated. In addition, although standardized nursing protocols were applied, variability in implementation and documentation may have introduced potential bias. Future multicenter prospective studies with larger samples and longer follow-up are warranted to further validate our findings.

## 5. Conclusion

In this study, the use of combined motivational psychological nursing and narrative nursing for CRF patients undergoing hemodialysis was effective and well-accepted by patients. This approach improved patient compliance and satisfaction, significantly alleviated negative emotions, reduced medical disputes, and enhanced the overall image of the hospital.

## Author contributions

**Conceptualization:** Ke Mei, Jun Gao, Ziqun Peng, Guohui Liu.

**Data curation:** Ke Mei, Jun Gao, Ziqun Peng, Ziang Zhang, Guohui Liu.

**Formal analysis:** Ke Mei, Ziang Zhang, Guohui Liu.

**Funding acquisition:** Jun Gao, Ziang Zhang, Guohui Liu.

**Investigation:** Ziang Zhang, Guohui Liu.

**Writing – original draft:** Ke Mei, Ziqun Peng, Ziang Zhang, Guohui Liu.

**Writing – review & editing:** Ke Mei, Ziqun Peng, Ziang Zhang, Guohui Liu.
